# Alterations in Platelet Alpha-Granule Secretion and Adhesion on Collagen under Flow in Mice Lacking the Atypical Rho GTPase RhoBTB3

**DOI:** 10.3390/cells8020149

**Published:** 2019-02-11

**Authors:** Martin Berger, David R. J. Riley, Julia Lutz, Jawad S. Khalil, Ahmed Aburima, Khalid M. Naseem, Francisco Rivero

**Affiliations:** 1 Centre for Atherothrombosis and Metabolic Disease, Hull York Medical School, Faculty of Health Sciences, University of Hull, HU6 7RX Hull, UK; mberger@ukaachen.de (M.B.); david.riley@hyms.ac.uk (D.R.J.R.); juliablutz@googlemail.com (J.L.); jawad.khalil@hyms.ac.uk (J.S.K.); ahmed.aburima@hyms.ac.uk (A.A.); 2 Department of Internal Medicine 1, University Hospital, RWTH Aachen, 52074 Aachen, Germany; 3 Leeds Institute for Cardiovascular and Metabolic Medicine, University of Leeds, LS2 9NL Leeds, UK; k.naseem@leeds.ac.uk

**Keywords:** adhesion, collagen, platelets, Rho GTPases, RhoBTB3

## Abstract

Typical Rho GTPases, such as Rac1, Cdc42, and RhoA, act as molecular switches regulating various aspects of platelet cytoskeleton reorganization. The loss of these enzymes results in reduced platelet functionality. Atypical Rho GTPases of the RhoBTB subfamily are characterized by divergent domain architecture. One family member, RhoBTB3, is expressed in platelets, but its function is unclear. In the present study we examined the role of RhoBTB3 in platelet function using a knockout mouse model. We found the platelet count, size, numbers of both alpha and dense granules, and surface receptor profile in these mice were comparable to wild-type mice. Deletion of *Rhobtb3* had no effect on aggregation and dense granule secretion in response to a range of agonists including thrombin, collagen, and adenosine diphosphate (ADP). By contrast, alpha-granule secretion increased in mice lacking RhoBTB3 in response to thrombin, collagen related peptide (CRP) and U46619/ADP. Integrin activation and spreading on fibrinogen and collagen under static conditions were also unimpaired; however, we observed reduced platelet accrual on collagen under flow conditions. These defects did not translate into alterations in tail bleeding time. We conclude that genetic deletion of *Rhobtb3* leads to subtle alterations in alpha-granule secretion and adhesion to collagen without significant effects on hemostasis in vivo.

## 1. Introduction

Activation of blood platelet receptors by thrombogenic proteins present in the extracellular matrix initiates a multistep process that involves numerous fine-tuned signaling events, leading to rapid platelet adhesion, degranulation, and aggregation [1]. Rho GTPases are molecular switches that play critical roles in platelet function, regulating the dynamics of the actin cytoskeleton, aggregation, secretion, spreading and thrombus formation [2]. Platelets contain several classical Rho GTPases which have been shown to influence platelet function and thrombosis primarily in mouse models [2,3]. Rac1 is required for lamellipodia formation and platelet spreading downstream of glycoprotein VI (GPVI) and protease activated receptors (PARs) and possibly also for secretion [4]. The role of Cdc42 is controversial due to conflicting observations from two different mouse models regarding its participation in filopodia formation, spreading, secretion, and aggregation [5,6]. Platelets lacking RhoA revealed a requirement of this protein for integrin activation, granule secretion and clot retraction [7]. No clear role has been identified for Rif [8], while RhoG appears important for integrin activation, aggregation and secretion in response to GPVI agonists [9,10].

RhoBTB proteins are atypical Rho GTPases and as such they differ from classical Rho GTPases in their regulation and/or domain architecture. More specifically, atypical Rho GTPases do not follow the classic cycle of activation and inhibition facilitated by guanine nucleotide exchange factors and GTPase activating proteins, but are regulated at protein expression levels or by specific protein-protein interactions [11]. RhoBTB proteins are characterized by a carboxyl terminal extension capable of assembling cullin 3-dependent ubiquitin ligase complexes [12]. Although their cellular roles are not fully elucidated, it is clear that unlike classical Rho GTPases these proteins bear no apparent relationship to direct remodeling of the cytoskeleton. RhoBTB proteins are implicated in tumourigenesis through regulation, among others, of the cell cycle and apoptosis (reviewed in [12]). RhoBTB3 additionally appears to be implicated in aspects of vesicle trafficking, such as retrograde transport to the Golgi and endosome to lysosome trafficking [13,14]. We have shown that RhoBTB3 deficient animals are characterized by a postnatal growth defect, reduced testis size in the males and deficient fertility [15]. All three members of the RhoBTB subfamily are present in platelets at mRNA levels [16] but the potential relevance of RhoBTB proteins for platelet function has not been investigated to date. Using a RhoBTB3 knockout (KO) model we extend our previous report by an in depth characterization of platelet function using a battery of conventional functional assays. Our data shows that the loss of RhoBTB3 is associated with altered alpha-granule secretion and a defect in collagen-mediated accrual, but otherwise this protein appears to be dispensable for hemostasis in vivo.

## 2. Materials and Methods

### 2.1. Reagents

Iscove’s Modified Dulbecco’s Medium (IMDM) was from Gibco/ThermoFisher Scientific (Loughborough, UK). Recombinant human erythropoietin and murine interleukin 3 were from PeproTech (London, UK). Phosflow Lyse/Fix Buffer and P-selectin were from BD Biosciences (Oxford, UK). Gly-Phe-Hyp-Gly-Glu-Arg (GFOGER) and collagen related peptide (CRP) were from Cambridge University (Cambridge, UK). Thrombin, ADP, fibrinogen, Gly-Pro-Arg-Pro-NH_2_, D-Phe-Pro-Arg-chloromethylketone (PPACK) and TRITC-conjugated phalloidin were from Sigma-Aldrich (Dorset, UK). Collagen reagent Horm was from Takeda (Osaka, Japan). Heparin sodium was from Leo Laboratories Limited (Berkshire, UK).

### 2.2. Cultivation of MKD1 Cell Line

The murine megakaryocytic cell line MKD1 clone G10 [17] was cultivated in IMDM supplemented with 10% fetal bovine serum, 2 mM l-glutamine, 0.15 mM monothioglycerol, 0.4 ng/mL human erythropoietin, 10 ng/mL murine interleukin 3 and antibiotics. Cultures were maintained at 37 °C in a humidified atmosphere containing 5% CO_2_.

### 2.3. Reverse Transcription Polymerase Chain Reaction (RT-PCR) 

Total ribonucleic acid (RNA) was isolated from MKD1 cells or mouse heart using a High Pure RNA isolation kit from Roche (Burgess Hill, UK) following the manufacturer’s instructions. RT-PCR was performed with a GoScript reverse transcriptase kit from Promega (Southampton, UK) using following set of *Rhobtb3*-specific reverse primers for the cDNA synthesis reaction: GTPaseR, 5′-TTCACTTGTCTTCTGATTTAAGGC-3′; E3R, 5′-ACTGTCAAAAATGTCCCAG-3′ and RhoBTB3R, 5′-TCACATGACTAAACAGCGACATTTCAG-3′. An aliquot of the cDNA synthesis reaction was used as template for a standard PCR with *Rhobtb3* primers RhoBTB3F (5′-ATGTCCATCCACATCGTGGCG-3′) and GTPaseR spanning exons 2–5 to yield a 618-bp product. Expression of *Gapdh* was determined as housekeeping control with following primers: forward, 5′-AGGCCGGTGCGAGTATGTC-3′; reverse, 5′-TGCCTGCTTCACCACCTTCT-3′. 

### 2.4. Experimental Animals

C57Bl/6 mice with a homozygous targeting of the *Rhobtb3* gene have been described elsewhere [15]. The animals were kept in the animal facility of the University of Hull using standard conditions. All animal work was performed in accordance with UK Home Office regulations, UK Animals (Scientific Procedures) Act of 1986, under the Home Office project license no. PPL 60/4024. For all experiments age-matched wild-type (WT) littermates were used as controls.

### 2.5. Preparation of Washed Platelets

Murine platelets were isolated as previously described [18]. Briefly, blood was taken by cardiac puncture into acid citrated dextrose (ACD) (113.8 mM d-glucose, 29.9 mM trisodium citrate, 72.6 mM NaCl, 2.9 mM citric acid, pH 6.4), centrifuged at 100× *g* for 5 min and the platelet rich plasma (PRP) was collected in a separate tube. Modified Tyrode’s buffer (150 mM NaCl, 5 mM HEPES, 0.55 mM NaH_2_PO_4_, 7 mM NaHCO_3_, 2.7 mM KCl, 0.5 mM MgCl_2_, 5.6 mM d-glucose, pH 7.4) was added to the blood and the procedure repeated to increase the platelet yield. The platelets were then pelleted at 800× *g* for 6 min, resuspended in modified Tyrode’s buffer and used for all consecutive experiments. 

### 2.6. Hematological Measurements

ACD-anticoagulated whole blood was diluted 1:20 in red blood cell lysis buffer (0.25 mM EDTA, 0.15 M NH_4_Cl, 0.01 M NaHCO_3_) for 1 min and 10 μL were transferred onto a Neubauer hemocytometer. White blood cells and platelets were counted in duplicate. Red blood cell counts and hematocrit were determined as described previously [19].

### 2.7. Flow Cytometry

PRP was prepared in sodium-citrate (110 mM trisodium citrate, pH 7.4). PRP was stimulated with CRP or ADP for 20 min at 37 °C in the presence of FITC-conjugated anti-P-selectin (BD Biosciences, Oxford, UK) and PE-JON/A (Emfret, Würzburg, Germany). Platelets were subsequently fixed and analyzed by fluorescence activated cell sorting (FACS) using an LSRFortessa cell analyzer (BD Biosciences, Oxford, UK). For receptor expression studies platelets were incubated with FITC-conjugated antibodies directed against surface membrane glycoproteins GP1b (CD42b), GPVI, integrin α_2_ (CD49b) (Emfret, Eibelstadt, Germany) and integrin α_IIb_ (CD41) (BD Biosciences, Oxford, UK). Receptor expression was also studied upon stimulation with 0.1 U/mL thrombin for 20 min at 37 °C in the presence of 10 µM Gly-Pro-Arg-Pro-NH_2_. Platelets were subsequently analyzed by FACS.

### 2.8. Platelet Aggregation and Adhesion

Platelet aggregation in response to agonists was recorded under constant stirring conditions (1000 rpm) for 4 min at 37 °C using Born aggregometry. For adhesion studies coverslips were coated overnight at 4 °C with fibrinogen, collagen, CRP or GFOGER at the concentrations indicated and blocked with heat denatured fatty acid free bovine serum albumin for 1 h before the experiment. Washed platelets were allowed to spread for 1 h, fixed with 4% paraformaldehyde (PFA), permeabilized with 0.3% Triton X-100 and stained with TRITC-labelled phalloidin. Platelets were imaged by fluorescence microscopy using a Zeiss ApoTome.2 equipped with an AxioCam 506 and a Zeiss Plan-Apochromat 63x NA 1.4 objective. Platelets were manually counted, and the surface coverage area was analyzed by thresholding using ImageJ.

### 2.9. Lumiaggregometry

Adenosine triphosphate (ATP) release was measured using CHRONO-LUME firefly luciferin/luciferase reagent (CHRONO-LOG, Havertown, PA, USA). Washed platelets (2.5 × 10^8^ platelets/mL) were incubated at 37 °C for 5 min in a CHRONO-LOG lumiaggregometer under non-stirring conditions. CHRONO-LUME was added for 2 min, followed by stimulation with thrombin under stirring conditions (1000 rpm). Secretion traces were recorded for 5 min. 

### 2.10. Electron Microscopy

Washed platelets were fixed in 0.1% glutaraldehyde in White’s saline (0.6 M NaCl, 5 mM KCl, 3.8 mM MgSO_4_, 4.5 mM Ca(NO_3_)_2_, 6.5 mM NaHCO_3_, 0.35 mM Na_2_HPO_4_, 0.19 mM KH_2_PO_4_, 0.5 mg phenol red) and processed as described elsewhere [20]. Thin sections were cut with a diamond knife on an ultra-microtome. Samples were visualized with a JEOL 2010 transmission electron microscope equipped with a Gatan Ultra Scan 4000 camera (JEOL Ltd, Tokyo, Japan).

### 2.11. Arterial Flow Experiments

Whole murine blood containing 40 µM PPACK was stained with 1 µM DiOC6 for 10 min at 37 °C. Blood was then perfused through 50 µg/mL collagen or 1 mg/mL fibrinogen coated capillary tubes at a shear rate of 1000 s^−1^ for 2 min and images of stably adhered platelets/thrombi were captured as previously described [18]. Thrombus volume was measured as previously described [21].

### 2.12. Tail Bleeding Assay

Mice were anesthetized with 5 mg/kg thiopental (Link Pharmaceuticals, Horsham, UK). The tail was cut off at 3 mm from the tip and immediately immersed in 37 °C saline (0.9% *v*/*w* NaCl). Bleeding time was monitored until hemostasis for up to 10 min.

### 2.13. Statistical Analysis

Experimental data was analyzed by GraphPad Prism v6.0 (La Jolla, CA, USA). Data are presented as means ± standard error of the mean (SEM) or standard deviation (SD) of at least 3 independent experiments. Normality was assessed by the Shapiro-Wilk test. Differences between groups were assessed using the Student’s *t*-test, Mann-Whitney U-test or analysis of variance (ANOVA) and statistical significance taken at *p* ≤ 0.05. 

## 3. Results

### 3.1. Rhobtb3 mRNA is Present in Mouse Megakaryocytes 

Despite extensive efforts with several commercial and custom-made antibodies, we were not able to detect RhoBTB3 protein in platelet and megakaryocyte lysates, which we attribute to poor antibody quality as well as low levels of the protein being expressed in platelets (data not shown). Of note, a literature survey shows that available antibodies fail to recognize any endogenous RhoBTB in fixed cells and tissues and very seldom in cell lysates [12]. Low protein levels and the fact that RhoBTB3 is a predominantly Golgi protein and very little Golgi is present in mature platelets may explain why platelet proteomics data fails to detect RhoBTB3 in mouse or human platelets [22,23]. 

**Figure 1:**
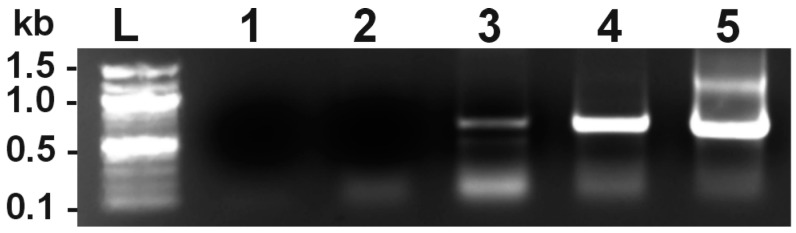
Expression of *Rhobtb3* in a mouse megakaryocyte cell line. RT-PCR was performed on RNA extracted from the mouse megakaryocyte cell line MKD1 (lane 3) or mouse adult heart (lane 4). The cDNA synthesis was done with a mix of reverse *Rhobtb3*-specific primers and the PCR with *Rhobtb3* primers spanning exons 2–5 to yield a 618-bp product. A cDNA synthesis with a reverse *Gapdh* primer was done on MKD1 RNA followed with a PCR with primers to yield a 530-bp product corresponding to the housekeeping gene *Gapdh* (lane 5). Lane 1 is a PCR control reaction with *Rhobtb3* and *Gapdh* primers and no template. Lane 2 is a PCR reaction with *Rhobtb3* primers, and the product of a cDNA synthesis performed with MKD1 RNA and *Rhobtb3* primers but without reverse transcriptase. L, ladder.

To verify the expression of *Rhobtb3* in the megakaryocyte cell lineage we extracted RNA from the embryonic stem cell derived murine megakaryocyte cell line MKD1 [17]. This approach was considered superior to using primary murine megakaryocytes in terms of amount of material and, more importantly, cell type homogeneity. Using RT-PCR with specific reverse primers we were able to detect *Rhobtb3* mRNA in MKD1 cells (Figure 1). The PCR reaction yielded the expected 618-bp product in both MDK1 and mouse heart cDNA as previously described [15]. We therefore conclude that *Rhobtb3* is expressed in cells of the megakaryocytic lineage. This result is consistent with the presence of RhoBTB3 encoding transcripts in mouse platelet transcriptomics studies [16].

### 3.2. Receptor Expression is Unaffected in RhoBTB3 Deficient Platelets

**Figure 2:**
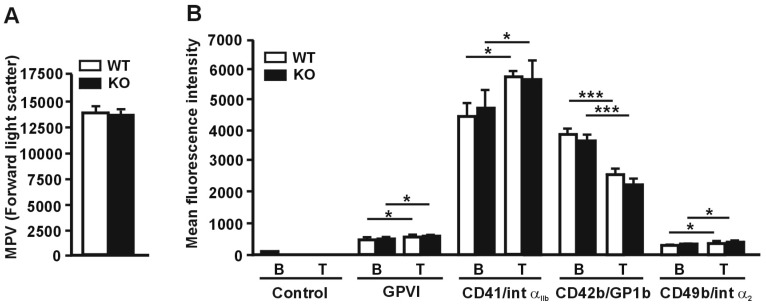
Relative size and receptor expression in RhoBTB3 deficient platelets. (**A**) Mean platelet volume (MPV) was measured by median forward light scatter height using flow cytometry. Data represents average ± SEM of 6 independent experiments. (**B**) Platelet surface receptors were determined by flow cytometry both in basal conditions (B) and upon stimulation with 0.1 U/mL thrombin for 20 minutes. Data represents average ± SEM of 6 independent experiments. * *p* < 0.05; *** *p* < 0.001; paired Student’s *t*-test between basal and stimulated conditions. No significant differences were observed between WT and KO platelets (non-paired Student’s *t*-test).

We hypothesized that RhoBTB3 may play roles in Golgi function in megakaryocytes during platelet formation, prompting us to study the effect of *Rhobtb3* gene disruption in platelet morphology and function in a RhoBTB3 KO mouse model characterized by our group previously [15]. Hematological evaluation of RhoBTB3 KO animals indicated that hematopoiesis is not affected, as evidenced by similar red blood cell, leukocyte and platelet counts to WT littermates (Table 1). The size of RhoBTB3 KO platelets was comparable to that of WT platelets as measured in the forward light scatter of flow cytometry experiments (*p* = 0.62, Student’s *t*-test) (Figure 2A).

When we assessed the expression of characteristic surface platelet receptors (GPVI, CD41, CD42b and CD49b) by FACS we found no significant alterations (Figure 2B). We also investigated whether platelet activation with thrombin would reveal any effect in the receptors’ behavior in RhoBTB3 KO platelets that could be related to a participation of this protein in vesicle trafficking events. Thrombin stimulation caused a modest but significant increase in the expression of GPVI and CD41 (18–22%) and CD49b (4–8%). A more profound decrease in the expression of CD42b (32–40%) was observed, due to cleavage and internalization of the GP1b/IX/V complex [24]. However, those effects were comparable in RhoBTB3 WT and KO platelets (Figure 2). We conclude that RhoBTB3 is dispensable for platelet production and surface receptor expression.

### 3.3. Secretion from Alpha Granules is Altered in RhoBTB3 Deficient Platelets

**Figure 3:**
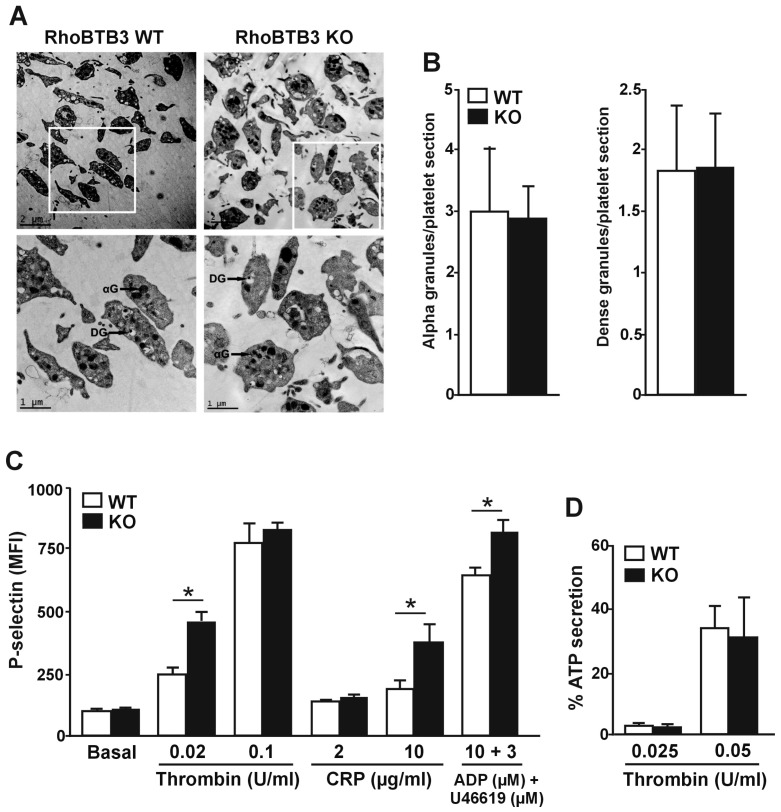
Ultrastructure and secretion in RhoBTB3 deficient platelets (**A**) Representative images of platelet ultrastructure of RhoBTB3 KO and WT mice. DG, dense granule; αG, alpha granule. (**B**) Quantification of the number of alpha and dense granules per platelet. Data was obtained from transmission electron micrographs. Only entire platelets were scored. Data represent average ± SD of 100–200 platelets from 3 independent preparations. (**C**) P-selectin expression (median fluorescence intensity, MFI) of either resting or stimulated platelets from 5 µL of whole blood with the indicated doses of thrombin, CRP, or a combination of ADP and U46619. The data represent the average ± SEM of 6 independent experiments. * *p* < 0.05; Mann-Whitney U-test. (**D**) ATP secretion upon thrombin stimulation. Washed platelets (2.5 × 10^8^ platelets/mL) were incubated at 37 °C in a CHRONO-LOG lumiaggregometer in the presence of CHRONO_LUME for 2 min, followed by stimulation with the indicated doses of thrombin. Secretion traces were recorded for 5 min and used to calculate the percentage of ATP secretion. The data represent the mean ± SEM of 3 independent experiments.

RhoBTB3 has been implicated in intracellular vesicle trafficking and therefore we hypothesized that granular morphology and degranulation might be defective in RhoBTB3 KO platelets [12,25]. We assessed the platelet ultrastructure by transmission electron microscopy to identify potential morphological alterations. Within a wide range of size and shape variability, we did not observe any difference between WT and KO platelets (Figure 3A). Alpha and dense granule distribution appeared not to be affected by ablation of *Rhobtb3* (alpha granules per platelet: 2.97 ± 0.43 in WT vs. 2.84 ± 0.21 in KO, *p* = 0.77; dense granules per platelet: 1.80 ± 0.12 in WT vs. 1.81 ± 0.16 in KO, *p* = 0.92) (Figure 3B). We set out to explore whether, despite a similar morphology, RhoBTB3 KO platelets have a defect in granule secretion. To monitor alpha-granule secretion, we induced platelet P-selectin expression by hemostatic agonists of varying potency (thrombin, CRP, and a combination of ADP and the thromboxane analog U46619). A trend towards increased response was observed with all agonists that reached statistical significance with 0.02 U/mL thrombin (*p* = 0.0152), 10 µg/mL CRP (*p* = 0.0152) and the synergistic combination of 10 µM ADP and 3 µM U46619 (*p* = 0.0411) (Figure 3C). Next we assessed dense granule secretion by ATP luminometry in response to varying doses (0.025 and 0.05 U/mL) of thrombin stimulation. None of the conditions tested revealed any significant difference in ATP secretion (Figure 3D).

### 3.4. RhoBTB3 Deficient Platelets Show Normal Integrin α_IIb_β_3_ Activation and Aggregation

**Figure 4:**
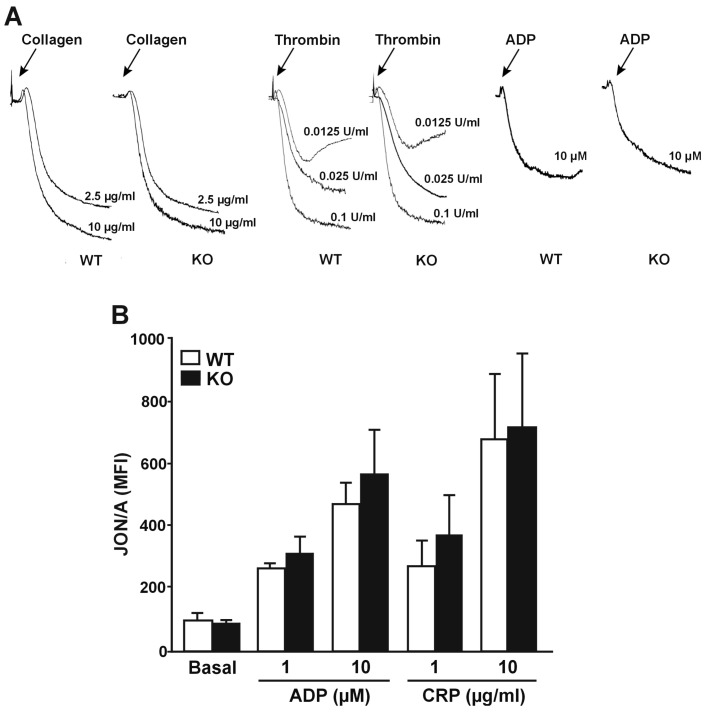
Normal aggregation and α_IIb_β_3_ integrin activation in RhoBTB3 deficient platelets. (**A**) Washed platelets (2.0 × 10^8^ platelets/mL) were stimulated with the indicated doses of collagen, thrombin or ADP and aggregation was recorded under constant stirring conditions (1000 rpm) for 4 min at 37 °C in a CHRONO-LOG aggregometer. Traces are representative of 3 independent experiments. (**B**) Integrin activation (median fluorescence intensity, MFI) upon platelet stimulation from 5 µL of whole blood for 20 min with the indicated doses of ADP or CRP and subsequent analysis on flow cytometry. The data represent the mean ± SEM of 3 independent experiments.

The potential effects of RhoBTB3 deficiency on integrin α_IIb_β_3_ activation were assessed indirectly by Born aggregometry and directly by the activation state-specific antibody JON/A by FACS. Collagen (2.5–10 µg/mL), thrombin (0.0125, 0.025 and 0.1 U/mL) and ADP (10 µM) all induced aggregation of washed platelets isolated from RhoBTB3 KO mice, which was of similar extent to that observed with WT platelets (Figure 4A). Using a more sensitive FACS approach RhoBTB3 KO platelets treated with a range of agonists ADP (1 and10 µM) and CRP (1 and 10 µg/mL) caused activation of α_IIb_β_3_ as evidenced by increased binding of JON/A. However, we were not able to detect any significant differences in the α_IIb_β_3_ activatory state between RhoBTB3 KO and WT platelets (Figure 4B). Therefore, RhoBTB3 appears to be dispensable for α_IIb_β_3_ activation and subsequent platelet aggregation.

### 3.5. Defective Accrual of Rhobtb3 Deficient Platelets on a Collagen Matrix under Arterial Flow

**Figure 5:**
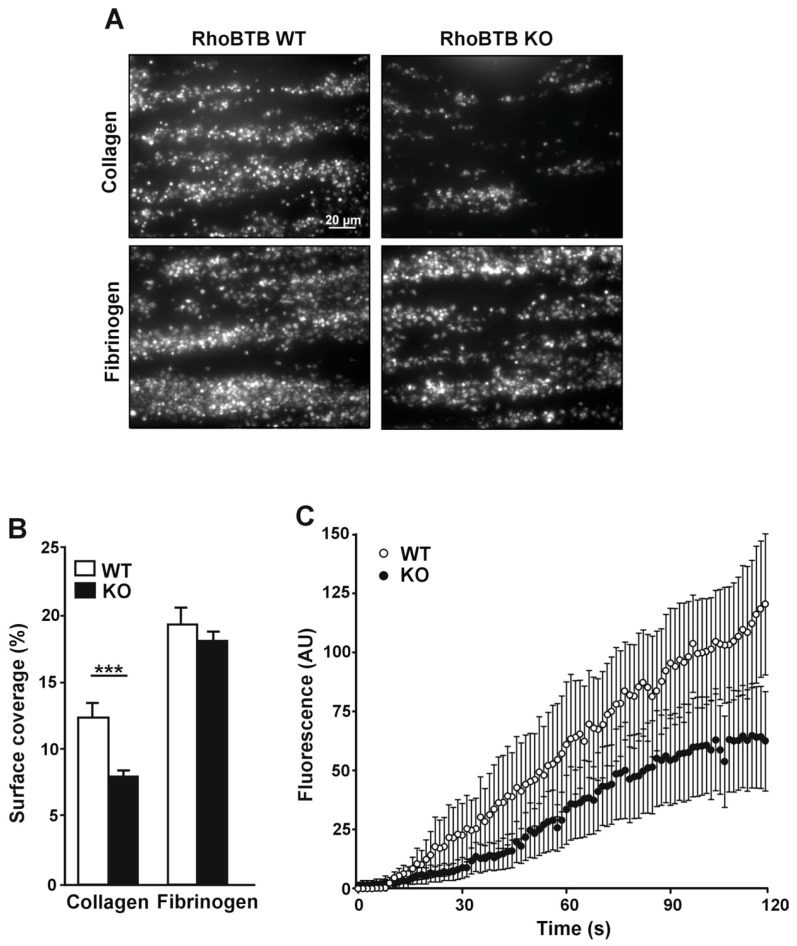
Behavior of RhoBTB3 KO and WT platelets on collagen or fibrinogen coated surfaces under flow. (**A**) Whole blood was stained with 1 µM DiOC6 for 10 min at 37 °C and perfused through 50 µg/mL collagen or 1 mg/mL fibrinogen coated capillary tubes at a shear rate of 1000 s^−1^ for 2 min. Representative images after 2 min are shown. (**B**) Quantification of surface coverage. Data are average ± SEM of images like those of panel A from 3 (fibrinogen) or 4 (collagen) independent experiments after 10 min of perfusion. *** *p* < 0.001, Mann-Whitney U-test. (**C**) Adhesion of platelets to collagen under flow as a function of time. Fluorescence intensity was calculated from images like those of panel A by thresholding using ImageJ. Data are average ± SEM of 4 independent experiments.

We next sought to explore platelets spreading in a physiologically relevant context under conditions of arterial blood flow. Perfusion of whole blood under arterial shear over a fibrinogen matrix led to platelet accrual and formation of a monolayer. Under these conditions the surface coverage with WT and KO blood at the end of the observation period was indistinguishable (18.12 ± 0.72% in the KO vs. 19.27 ± 1.29% in the WT, *p* = 0.44). In contrast we observed that on collagen less platelets from KO blood adhered compared to WT resulting in a reduced surface coverage at the end of the observation period (8.9 ± 0.7% in the KO vs. 13.8 ± 0.8% in the WT, *p* < 0.0001; Mann-Whitney U-test) (Figure 5A,B). To investigate the dynamics of adhesion to collagen and thrombus volume accrual under flow we plotted the fluorescence intensity as a function of time and found that with RhoBTB3 KO platelets adhesion and thrombus volume increase occurred at a lower rate during the complete period of observation (area under the curve: WT 5082 AU vs. KO 2848 AU) (Figure 5C). In summary, genetic deletion of *Rhobtb3* led to a reduced adhesion to collagen under arterial flow conditions.

**Figure 6:**
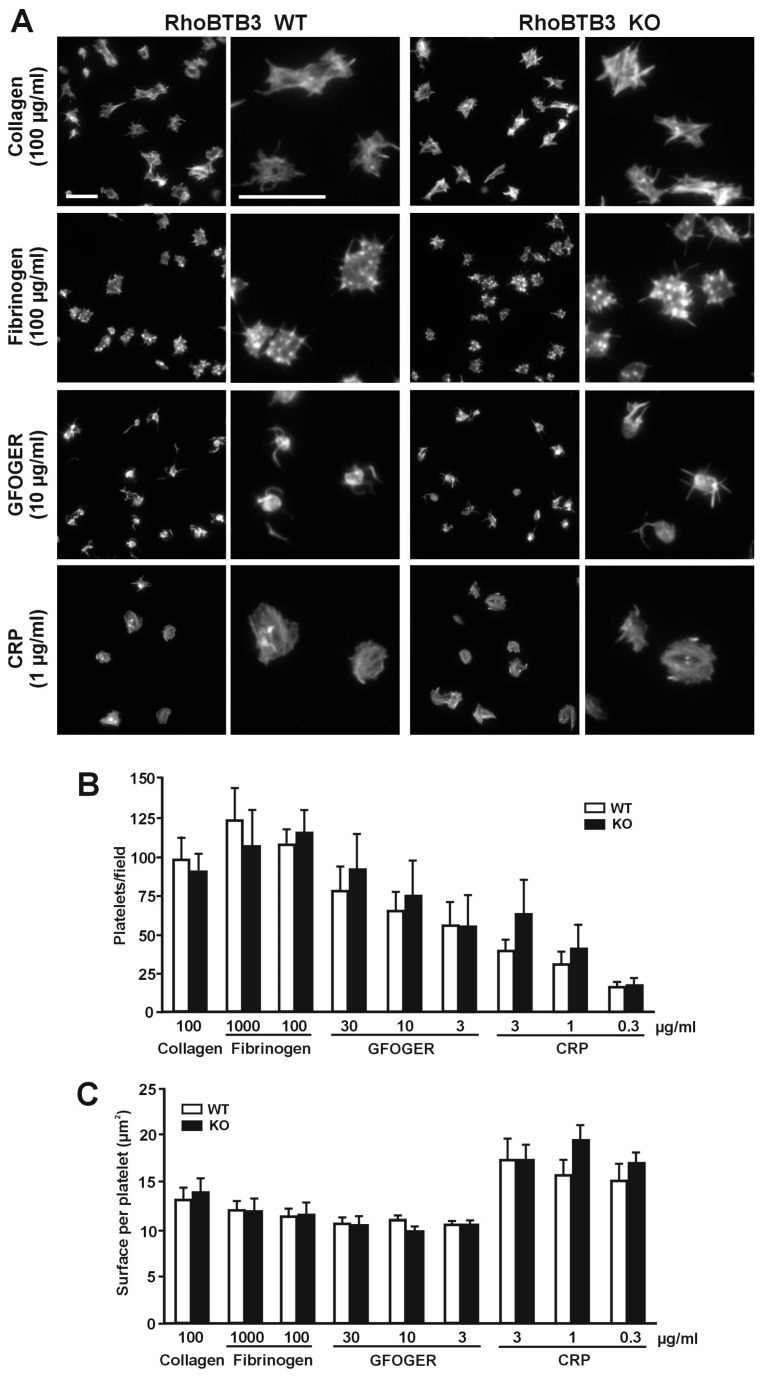
Unimpaired spreading of RhoBTB3 deficient platelets. (**A**) Adhesion of RhoBTB3 KO and WT platelets to glass coverslips coated with the indicated concentration of collagen, fibrinogen, GFOGER, or CRP. Adherent platelets were fixed with 4% PFA, permeabilized with 0.3% Triton X-100 and stained with TRITC-phalloidin. Platelets were visualized with a fluorescence microscope and images of random areas were acquired. For each phenotype the right column shows examples of platelets at higher magnification. Scale bars represent 5 µm. (**B**) Number of platelets adhering to the indicated concentrations of collagen, fibrinogen, GFOGER, or CRP. 10 fields each 12,500 µm^2^ from 5–10 independent experiments were counted per condition. Data represents average ± SEM. No significant differences were found between WT and KO platelets for any condition (Mann-Whitney U-test). (**C**) Surface coverage per platelet calculated by thresholding using ImageJ. Data represent average ± SEM from 5–10 independent experiments and 250–1000 platelets per condition for each experiment. No significant differences were found between WT and KO platelets for any condition (Mann-Whitney U-test).

To narrow down the adhesion defect observed under conditions of arterial flow, we investigated platelet adhesion and spreading on surfaces coated with collagen (100 µg/mL) or fibrinogen (1000 and 100 µg/mL). Slightly, although statistically not significantly, more WT platelets per observation field adhered on fibrinogen (123.3 ± 21.0 on 1000 µg/mL and 107.4 ± 10.2 on 100 µg/mL) than on collagen (98.3 ± 14.3). However, there was no difference in the number of platelets adhering to either surface between the WT and the genetically modified mice (105.6 ± 24.1, 113.9 ± 15.0 on fibrinogen and 89.3 ± 12.7 on collagen) (Figure 6A,B). Detailed examination of the spread platelets revealed that both WT and KO platelets covered a slightly (but not significantly) larger surface on collagen (13.01 ± 1.32 µm^2^ in the WT vs. 13.71 ± 1.65 µm^2^ in the KO) than on fibrinogen (11.20 ± 0.92 µm^2^ in the WT vs. 11.31 ± 1.55 µm^2^ in the KO for 100 µg/mL; similar values for 1000 µg/mL) (Figure 6C). On collagen platelets displayed prominent stress fibers and the cells often appeared stretching along matrix fibers, whereas on both concentrations of fibrinogen (only 100 µg/mL shown as an example in Figure 5A) platelets showed abundant filopods and actin nodules. No noticeable differences between WT and KO platelets were apparent in the morphology in any of the matrices.

Platelet adhesion to collagen is mediated by two different receptors, GPVI and α_2_β_1_ integrin. To narrow down the accrual defect we observed under flow conditions to a potential defect in one of those receptors we investigated adhesion and spreading to various concentrations of peptides that specifically bind to one receptor, GFOGER (for α_2_β_1_) and CRP (for GPVI) (Figure 6). In general, less platelets adhered on GFOGER compared to collagen, in a matrix concentration dependent manner, down to approximately 50% at the lowest matrix concentration. The trend was similar in both WT and KO platelets. Significantly less platelets (16.4 ± 2.7 in the WT vs. 17.6 ± 4.4 at the lowest matrix concentration) adhered on CRP compared to the respective spreading data on collagen (*p* = 0.001) (Figure 5B). Surface coverage was slightly lower on GFOGER (10.52 ± 0.25 µm^2^ in the WT vs. 10.41 ± 0.31 µm^2^ in the KO at the lowest matrix concentration) and higher on CRP (14.95 ± 1.97 µm^2^ in the WT vs KO 16.90 ± 1.10 µm^2^ in the KO at the lowest matrix concentration) compared to collagen, but these differences did not reach statistical significance (Figure 6C). While platelets on CRP morphologically resembled the ones on collagen, on GFOGER they looked more discoid and displayed long, thick and sometimes branched filopods (Figure 6A shows examples at intermediate matrix protein concentrations). No significant differences between WT and KO were found in the platelet numbers, surface covered, and morphology on any of the two receptor-specific matrices at any of the concentrations tested.

**Figure 7:**
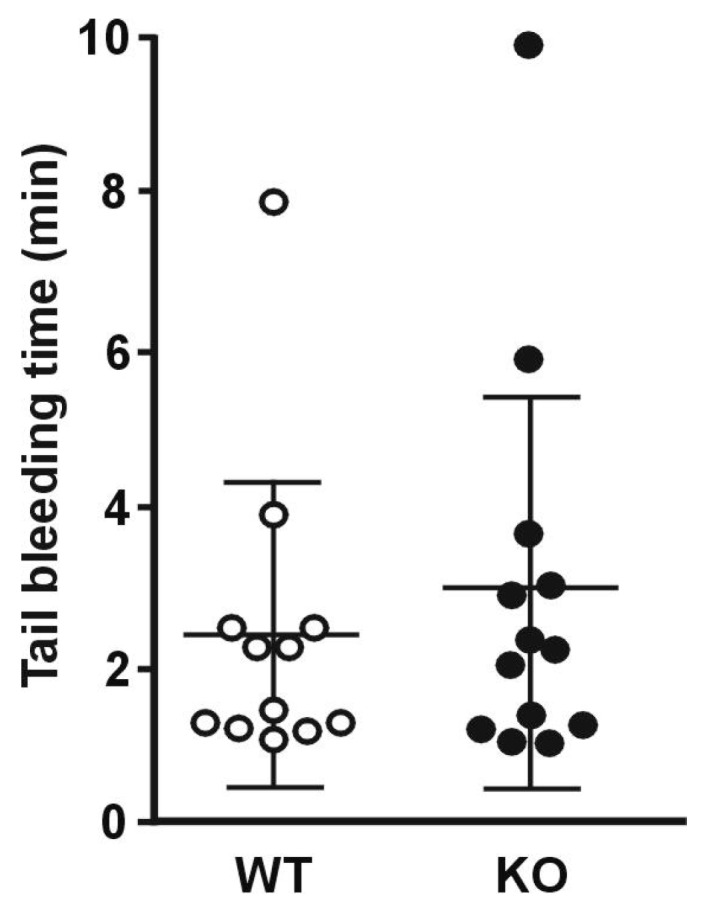
Tail bleeding time. Tests were performed by cutting off 3 mm of the tail tip and placing the tail in 37 °C saline. The time until hemostasis was recorded for up to 10 min and re-bleeding monitored for 60 s beyond hemostasis. Data represent average ± SD of 12–13 animals.

Finally, to evaluate the influence of *Rhobtb3* deletion on hemostasis, tail bleeding time was examined (Figure 7). Both RhoBTB3 KO and WT animals showed a comparable average bleeding time (KO 3.06 ± 2.48 vs. WT 2.51 ± 1.90; *p* = 0.97, Student’s *t*-test).

## 4. Discussion

Recent studies in animal and pharmacological models have significantly contributed to elucidate the roles of Rho GTPase signaling in platelet function. Although we still lack a clear picture, these studies have revealed the participation of major Rho GTPases, such as Rac1, Cdc42, and RhoA, as well as some of their effectors and regulators in various key platelet biology processes [2,3]. Comparatively little is known about the roles of atypical Rho GTPases in general, and virtually nothing in platelets. Here we contribute to our understanding of the potential relevance of RhoBTB3 using a KO mouse model for an in depth characterization of platelet function. The salient features of the genetic deletion of *Rhobtb3* are increased alpha-granule secretion and reduced accrual to collagen under low arterial shear conditions in the absence of any other overt morphological or functional alteration.

RhoBTB3 is itself a substrate for the cullin 3-based ubiquitin ligase complexes it helps recruit, and therefore it does not appear to accumulate [26]. Platelets possess an active proteasome-dependent degradation machinery [27], which linked to the fact that RhoBTB3 is a predominantly Golgi protein and very little Golgi is transmitted to platelets during thrombopoiesis [28] would explain the undetectable levels of this protein in mature platelets with the available tools. Nevertheless, RhoBTB3 may play roles in Golgi function in megakaryocytes during platelet formation, therefore any defect observable in platelets would be mainly the result of a qualitatively defective platelet biogenesis [29]. We exclude any quantitative defect in hematopoiesis since RhoBTB3 deficiency does not affect the production of platelets and other blood cells as shown by unaltered blood counts and platelet size.

RhoBTB3 has been shown to specifically interact with Rab9, which localizes to late endosomes and is required for lysosome biogenesis [13,30]. Loss of Rab9 or its effectors RhoBTB3, TIP47 and GCC185 results in mis-sorting of mannose-6-phosphate receptors to lysosomes [13]. Interestingly, Rab9 also interacts with BLOC-3 (biogenesis of lysosome-related organelles complex-3). Mutations in BLOC-3 have been identified in patients with Hermansky-Pudlak syndrome, who suffer from bleeding due to defective biogenesis of lysosome-related organelles such as dense granules [31]. We have not noticed any morphological defects in dense and alpha granules while also dense granule secretion appeared to be unaffected. However, we observed an increased reactivity in RhoBTB3 deficient platelets by P-selectin exposure upon stimulation with various agonists that was not accompanied by increased α_IIb_β_3_ activation. It is not unusual that a defect in alpha granules does not affect α_IIb_β_3_ as reported in storage pool deficient platelets from humans, which do not necessarily show a platelet aggregation defect in vitro [32]. RhoBTB3 apparently affects solely alpha-granule secretion and this effect might be traced back to a role of RhoBTB3 in vesicle trafficking during platelet biogenesis [12].

Similar phenotypes have been described before that link the observation of increased P-selectin expression to decreased adhesion to collagen. In a double KO for multimerin and alpha-synuclein Reheman et al. found increased levels of P-selectin upon thrombin stimulation accompanied by a decreased accrual on collagen under flow [33]. In the original phenotypical description of the Cdc42 KO mouse an approximately 10% decrease in percentage surface coverage on collagen was reported, while P-selectin expression was found increased [5]. Interestingly, in a double KO mouse of the related actin-binding proteins cortactin and its homolog hematopoietic lineage cell-specific protein 1 (HS1) the only salient defect was impaired accrual on collagen under high shear rates [34]. A fraction of RhoBTB3 localizes to early endosomes [26], where it may interact with Hrs (hepatocyte growth factor-regulated tyrosine kinase substrate) [14], a subunit of the endosomal sorting complex required for transport-0 (ESCRT-0) that captures ubiquitinated membrane proteins and mediates their recycling and retrograde trafficking [35]. Receptor recycling in general, and integrin recycling in particular, remains poorly understood in platelets. Different integrins follow distinct recycling mechanisms and routes [36] and a recent study has showed that disturbed recycling of integrin α_IIb_β_3_ with an inhibitor of clathrin-mediated endocytosis impaired spreading on fibrinogen [37], highlighting the importance of integrin recycling in platelets. Interestingly, we observe alterations in the adhesion to collagen, but not to fibrinogen, under flow conditions. We speculate that despite unaltered collagen receptor numbers α_2_β_1_ and/or GPVI may be sub-functional due to delayed turnover, impaired recycling, or impaired signaling. In this respect signaling through GPVI involves the Fcγ receptor as well as non-receptor tyrosine kinases of the Src family whose localization and signaling activities are tightly regulated by endocytic trafficking in various cell types [38,39].

In summary, we show that the loss of RhoBTB3 is associated with altered alpha-granule secretion and a defect in collagen-mediated accrual, which might be a testimony of the roles of this protein in vesicle trafficking processes during platelet biogenesis. Despite these alterations, bleeding time is not affected, making RhoBTB3 dispensable for hemostasis.

## Figures and Tables

**Table 1:** Hematology features of RhoBTB3 deficient mice. Counts were assessed with a Neubauer counting chamber. Hematocrit is expressed as percentage fraction of total. Data represents the average ± SD from 10 (hematocrit) or 11 (cell counts) animals of each genotype. *P* values are calculated from Student’s *t* tests.

	RhoBTB3 WT	RhoBTB3 KO	*p* Value
Red blood cells (µL^−1^)	9,842,237 ± 1,414,027	9,994,156 ± 1,953,946	0.84
White blood cells (µL^−1^)	7923 ± 3021	7464 ± 2796	0.72
Platelets (µL^−1^)	951,602 ± 407,511	847,438 ± 163,831	0.46
Hematocrit	47 ± 11	46 ± 15	0.92
